# Incarceration as a catalyst for worsening health

**DOI:** 10.1186/2194-7899-1-3

**Published:** 2013-10-24

**Authors:** Lauren Brinkley-Rubinstein

**Affiliations:** Department of Human and Organizational Development, Vanderbilt University, 230 Appleton Place, Peabody #90, Nashville, TN 37203 USA

## Abstract

The primary aim of this paper is to explicate the mechanisms through which incarceration affects health. Guided by theories that emphasize the compounding nature of inequality and with a focus on those that are disproportionately impacted by the drastic increase of incarceration over the last three decades, an exploration of these mechanisms is undertaken. This investigation provides a better understanding of the issues that are faced by incarcerated individuals in the incarceration environment, after release, and via macro-level policy. Finally, a hypothetical heuristic framework is presented that illustrates the ways in which incarceration affects individual, family and community level health. Implications for policy intervention programs and future research that serve to address diminished health among incarcerated populations are discussed.

**Electronic supplementary material:**

The online version of this article (doi:10.1186/2194-7899-1-3) contains supplementary material, which is available to authorized users.

The incarceration rate has risen steeply in the United States over the last several decades, increasing by 397% between 1980 and 2011 (Carson and Sabol 2012; National Institute of Justice 1984). In 2011, there were a total of 6.98 million people in the criminal justice system, including 2.17 million in jails or prisons and 4.81 million on probation or parole (Glaze 2012). Drucker (2011) suggests that if the population of incarcerated individuals were likened to a city, this city would be the second largest in the United States. In addition to these staggering statistics, each year, on average for the last 25 years, more than 10 million people are arrested. In 2009 alone, there were more than 13,687,000 people arrested leading to over 600,000 new prison inmates (Federal Bureau of Investigation 2010).

The causes of this extreme increase in the incarceration rate have been explored extensively across a number of disciplines. However, the factors that have led to the colossal rise in the number of incarcerated individuals are difficult to isolate. Importantly, the social context, both at the policy-level and micro-individual-level, has been widely cited as contributing to the surge in the incarcerated population (Alexander 2010; Drucker 2011; Mauer and Chesney 2002). Intersecting categories of disadvantage accounts for much of the relationship between mental health and incarceration (Draine et al. 2002). Mental health policy shifts, the closure of public mental health hospitals and a lack of political will to create alternative community support systems created a drastic reduction in available psychiatric inpatient beds and prohibition of admission to private hospitals for mental health patients (Lamb and Weinberger 2005). This resulted in an increased population of individuals living with untreated mental illness who are also increasingly likely to engage in “illegal behaviors” and, thus, become incarcerated. Additionally, policies related to substance abuse, such as those that culminated in the War on Drugs, led directly to an increase in drug-related arrests and the creation of harsher, more punitive laws regarding drug use (Boutwell and Rich 2004; Lurigio and Swartz 2000; Drucker 2011). Research demonstrates nearly two-thirds of the increase in the federal prison population was due to stricter, more mandatory and determinant sentencing for drug offenses rather than an actual increase in crime (Mauer and Chesney 2002; Wacqant 2010).

Concomitant with the War on Drugs was what Wacqant (2010) refers to as the “workfare revolution” that culminated in the Personal Responsibility and Work Opportunity Act (PRWOA) in 1996. These two simultaneous seismic policy shifts resulted in “hyperincarceration” that has disproportionately impacted low-income African Americans (Wacqant 2010). Incarceration rates among African Americans are much higher than their White counterparts. African American men specifically are incarcerated at a rate that is 650% greater (Sabol and Couture 2008). Strikingly, African Americans and Whites have nearly the exact same rate of drug use (7.4% for African Americans and 7.2% for Whites), but African Americans constitute almost 63% of drug arrests and more than 80% of drug possession arrests despite constituting only 13% of the total population (Fellner 2008,2009). Subsequently, the Bureau of Justice Statistics has projected that one in every three African American males is likely to go to jail or prison in his lifetime (Bonczar 2003). This disproportionate rate of incarceration, thus, affects many areas of one’s life including employment, education, and, most relevant herein, overall well being and health (Alexander 2010; Drucker 2011).

Those who are most likely to experience incarceration, such as African Americans, often have pre-existing disproportionately high rates of many chronic and infectious diseases due to the many other social determinants of health that differentially affect at-risk populations (Williams et al. 2010; Adler 2007). Extant scholarship has explored the multiple mechanisms of incarceration that may also have a detrimental effect on health (de Viggiani 2007). However, whereas previous research has explored and identified a link between social conditions and health, a clear and extensive model theorizing the mechanisms of incarceration, their connection to worsened health, and exacerbation of health disparities among the most impacted populations is missing from the literature (Link et al. 2010; Williams et al. 2010; Adler and Stewart 2010). In fact, most frameworks elucidating the social determinants that negatively effect health omit incarceration altogether. Only the World Health Organization’s Model of the Impact of the Social Determinants of Health mentions social exclusion, but it does not fully elaborate or define this facet of the model (Commission on Social Determinants of Health (CSDH). 2008).

Thus, this paper’s overall goal is to present the mechanisms through which incarceration exacerbates the conditions of an already medically disenfranchised population and contributes to a diminished health status of individuals, families and entire communities most impacted by hyperincarceration. Therefore, this paper’s specific aims are to: 1) present a theory that underpins the relationship between health and incarceration via a heuristic framework that hypothesizes how incarceration affects community, family and individual health, and thus, exacerbates health disparities; 2) elaborate on the specific mechanisms through which incarceration directly and indirectly deteriorates and impacts health via stress-producing circumstances that are imposed, enforced, and reinforced through the present day paradigm of criminal justice, and 3) to elucidate the policy, programmatic, intervention and future research implications that are necessary to address the effects of incarceration on health, and thus, address health disparities among those who are most likely to experience incarceration. However, first, a review of the research that examines the impact of incarceration on health is presented. This is necessary in order to better understand the relationship between health and incarceration.

## Literature review

The extant literature that explores the link between health and incarceration can be classified into three distinct categories. The first examines the incarcerated experience and health. This category explores the relationship between mental and physical health and incarceration and the provision of healthcare in correctional facilities. Related is the second category that includes research that explores the link between incarceration and actions that may also influence health, such as risky sexual behavior and substance use. The third and final category includes scholarship that investigates the post-release transition and well being. This research focuses on post-release barriers to community reintegration and the sustained effects that they may have on health.

## The incarcerated experience and health

Research has established a connection between incarceration and health. This is due, at least in part, to the potential for prisons and jails to amass individuals who are most at risk for accumulated disparities such as a high prevalence of experiencing violence, substance abuse, mental health issues and infectious and chronic diseases (Heron et al. 2009). Rates of HIV infection are four to six times higher, and one in three incarcerated individuals are estimated to have hepatitis C (Centers for Disease Control 2012; Maruschak 2006). About 4.2% of all tuberculosis cases occur in correctional facilities while less than 1% of the American population is incarcerated at any given time (Centers for Disease Control 2010; Schmitt et al. 2010). Additionally, Binswanger et al. (2009) found that those incarcerated in jails and/or prisons have a higher likelihood of experiencing hypertension, asthma, arthritis, and cervical cancer than their non-incarcerated counterparts. Prince (2006) analyzed hospital and prison administrative records and found that individuals who were diagnosed with schizophrenia who had a history of incarceration in New York City were more likely than their non-incarcerated peers to have a higher number of previous hospital stays, visits to the emergency room and re-hospitalization within three months of being discharged from the hospital. Research has been conducted that investigates the mortality rates of inmates compared to those in the general population. Studies have shown that the mortality gap narrows for some populations, in incarceration settings demonstrating the importance of routinized healthcare provision. African American inmates’ rate of mortality is lower compared to the general African American population, whereas Whites either have a higher or an unchanged mortality rate compared to their non-incarcerated White counterparts (Patterson 2010; Rosen et al. 2011; Spaulding et al. 2011). However, several studies have shown that incarceration is associated with decreased mortality among individuals post-release (Binswanger et al. 2011; Binswanger et al. 2007; Calcaterra et al. 2012). New findings from Patterson (2013) illustrate that each additional year in prison produced a 15.6% increase in the likelihood of death for parolees, translating to a 2-year decline in life expectancy for each year served in prison (Patterson 2013).

In addition to impacting physical health, research has also been conducted that illustrates incarceration’s impact on mental health. Previous findings indicate that imprisonment is independently associated with emotional reactions, such as anxiety, and that multiple incarcerations seem to elicit even stronger detrimental emotional reactions (Blanc et al. 2001; Schnittker et al. 2012). Incarcerated populations also have disproportionately high levels of various mental health issues such as depression and antisocial personality disorders (Fazel and Danesh 2002; Wilper et al. 2009) and post-release many inmates have a high rate of psychiatric disorders that may have gone undiagnosed (Mallik-Kane and Visher 2008). Finally, based on in-depth life interviews with individuals who served an average 19 years in a correctional institution, Liem and Kunst (2013) theorize that those who experience long-term incarceration may suffer from post-incarceration syndrome, which they likened to post traumatic stress disorder.

Compounding the health issues already faced by many inmates is the fact that healthcare infrastructure in correctional facilities can create barriers that limit access to medical care (Magee et al. 2005). Hatton et al. (2007) investigated the specific issues related to healthcare access in jails and found that errors caused by the facility itself, hygiene issues, mandatory requirement of co-payment, delay in obtaining needed medications, side effects from medications, administration of wrong medications, medications stopped by mistake and allergic reactions to medications were common and often influenced the health of inmates negatively. Thus, existing research repeatedly demonstrates the compounding impact of the incarceration on the physical and mental health of prisoners, both while serving their sentence and following release.

## The link between incarceration and engagement in risk behavior

Research has also explored the risk behavior of individuals who are prone to experience incarceration and how it influences the likelihood of re-incarceration and health status. A large focus of this literature examines the role of drug use, the nature of drug use for incarcerated populations, and the relationship between drug use and infectious diseases, such as HIV (Horton 2011). Studies have examined post–release sexual risk behavior and found that those who have a steady, long-term partner prior to incarceration are less likely to be inconsistent condom users, have sex while high on drugs and/or using alcohol, use marijuana daily or carry weapons during illegal activity immediately after release (Ramaswamy and Freudenberg 2010). Catz et al. (2012) also found that partners’ perceptions that being released from prison increases sexual desirability, partners’ negative condom attitudes, depression, strong desires for sex and/or substance use and HIV disclosure-related fears of rejection act as barriers to risk reduction after release from prison.

## Post-release transition and reintegration into the community

The final category of incarceration and health includes research that focuses on the post-release experience of former inmates and how reintegration into their home community affects access to healthcare and successful reintegration. They include issues related to micro, individual factors and macro-level policy such as inability to find a job or job training, issues related to medications (for those who are already ill), finding housing and shelter, administrative or bureaucratic barriers in obtaining services, lack of emotional support from both peers and professionals, issues with medical care including obtaining insurance such as Medicaid, transportation, and lack of availability of social services (Petersilia 2008; Sowell et al. 2001). Additionally, Rotter et al. (2005) posit that the experience of incarceration may force inmates to adapt to the prison environment by adopting a hyper masculine “inmate code.” This adaptation includes rules and values such as not reporting violations and not appearing weak within the prison walls. These attitudes, however, manifest and persist even after release and can cause confrontational behavior that may hinder successful reintegration and lead to re-incarceration.

## The mechanisms through which incarceration impacts health

**Figure 1:**
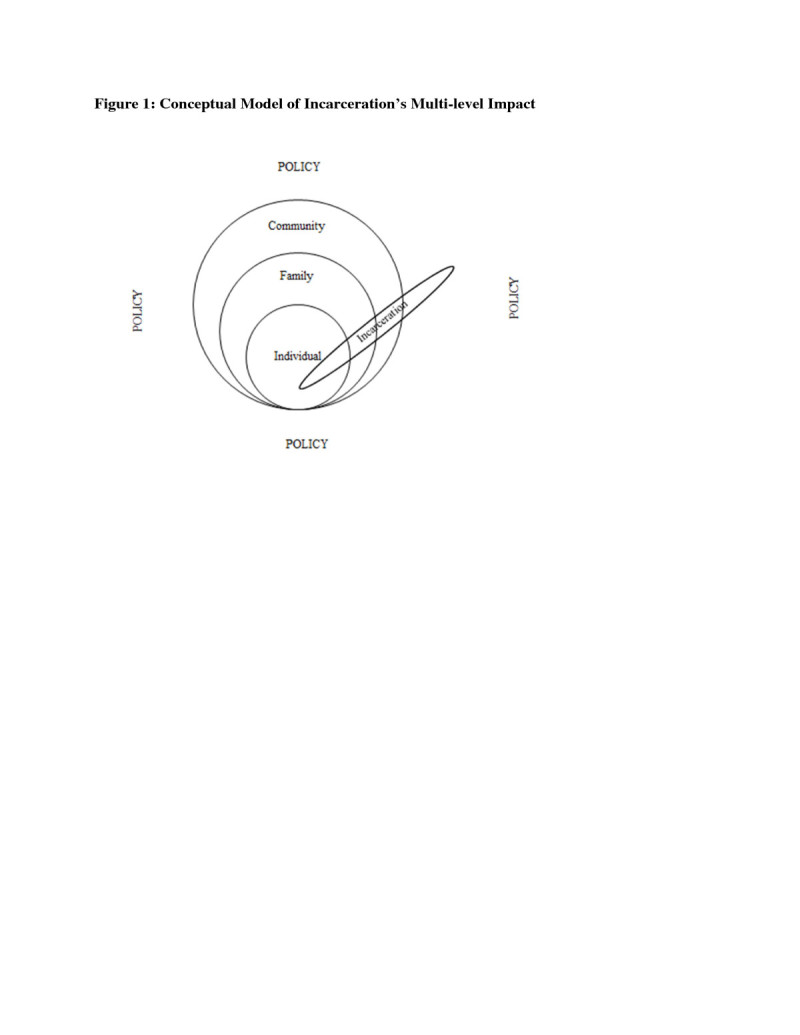
**Conceptual model of incarceration’s multi-level impact.**

**Figure 2:**
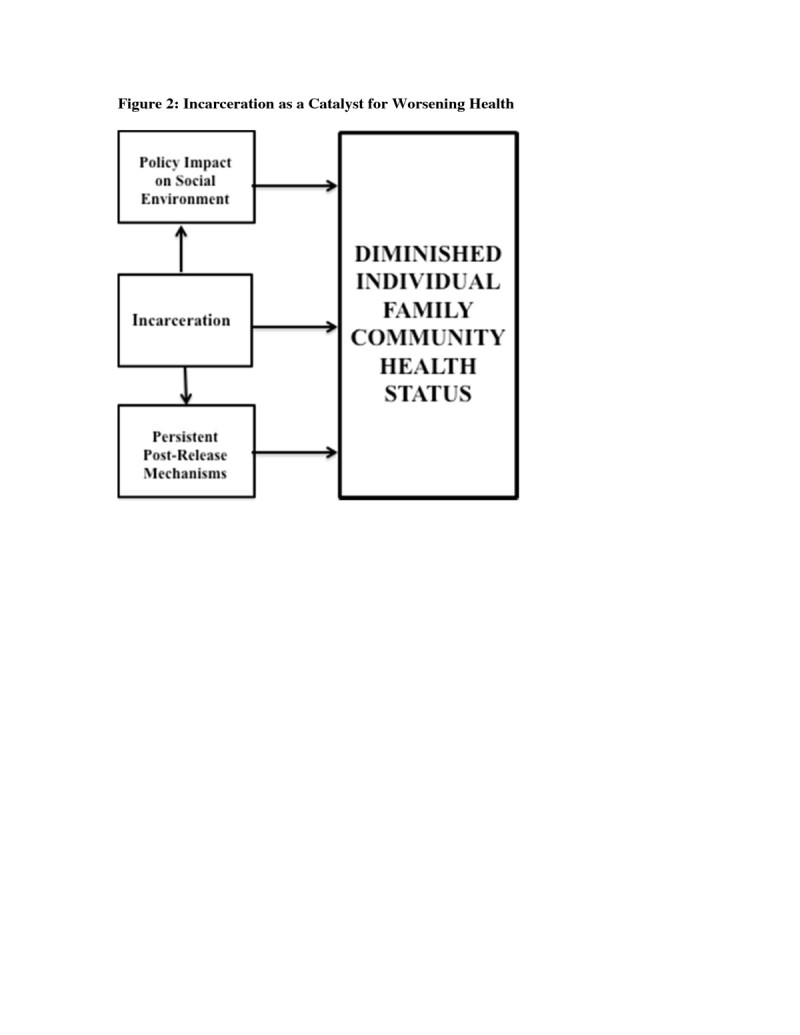
**Incarceration as a catalyst for worsening health.**

The remainder of this paper will explore ways in which incarceration directly and indirectly affects health. Figure 1 is a visual depiction of the crosscutting nature of incarceration on communities, families and individuals and serves as the conceptual grounding for the heuristic framework that is proposed. As such, it illustrates that incarceration has a multi-level impact that affects all realms of one’s life. Figure 2, then, is a heuristic path model elucidating how incarceration acts as a catalyst for worsening health. More specifically, this model presents a hypothetical path via which incarceration deleteriously affects multiple levels of health. More specifically it theorizes that incarceration affects the health of individuals, families and communities via the incarceration experience, worsening social conditions post-release and macro-level policy.

## Theoretical underpinnings

The conceptual and heuristic framework for the current study is guided by a number of inter-related theories that illustrate the cumulative effects of stressful and negative life events imposed via incarceration. They include intersectionality theory (Andersen and Collins 1998; Collins 2000; McCall 2005), which seeks to explain how social and cultural classifications (such as gender, race, class, ability and other axes upon which individuals build their identity) interact simultaneously to contribute to inequality; life course theory (Berkman 2009), relevant to exploring the longitudinal and continual impact of the incarceration experience; weathering (Geronimus and Thompson 2006), a conceptualization of aging in which vulnerable and at risk populations experience depreciated health because they have more severe and more recurrent experiences with societal and economic hardship than that experienced by other groups; and the social ecological model (Bronfenbrenner 1979; Rappaport 1981) which has a focus extending beyond individuals, taking a crucial stance that shifts responsibility for reducing health inequalities away from individuals onto the environmental factors and systems in which they are situated. Critical to each of these frameworks is the need to focus on the societal, policy, community, family and individual level rather than *just* micro-level behaviors. To date, the focus of most policies and research related to incarceration has been on the outcomes affiliated with behaviors, absent of considerations of the sociopolitical contexts that may impact individual decision-making. Thus, attention must be paid to the influence of macro-scale variables (e.g. drug law policies) and how societal conceptualizations of behavior affect an individual’s construction of attitudes and behavior over the life course. Furthermore, the interaction between individual and societal norms must be better understood in order to more comprehensively address the means through which incarceration intensifies health disparities longitudinally.

A long-term approach that takes into account the multidimensional nature of disparity is necessary to exploring the sustained and continual effect of the incarceration experience. A nuanced view of incarceration’s impact extends the existing literature because it assumes that the effect of incarceration is not temporary and limited to the time of imprisonment. Instead, incarceration has the ability to cascade into each area of one’s life and, as such, can affect individuals and communities on multiple levels (e.g. individual, family, community) and for extended durations. Additionally, those who are most likely to be incarcerated are also more likely to come from impoverished backgrounds, to have been victims of crime, live in violent, low-resource neighborhoods, and to have lower levels of educational attainment (Travis and Crayton 2009). Therefore, individuals who are most at risk of incarceration are already more likely to have lower levels of self-rated health, less access to medical care and health insurance, and lower quality of care (Veenstra 2011). These issues related to access and standards of care can compound to further exacerbate health disparities. Incarceration’s impact on health begins with the incarceration experience itself, is followed by post-release setbacks and has foundations in policy that restricts access to various rights, including employment and housing.

## Incarceration as a catalyst for worsening health

In this section, the ways in which incarceration negatively impacts health are explained in more detail by focusing on the specific mechanisms of incarceration that affect health via the incarceration environment, after release, and on the policy level. Although each of these mechanisms has a separate and distinct influence on health, it is essential to develop an understanding of the cumulative, continual and intersectional impact of the stressors and inequalities that are first experienced inside the walls of a prison or jail. It is also particularly important to explain the mechanisms through which incarceration negatively affects health not only for individuals but also for families and, eventually, communities. Table 1 identifies the main variables of interest—each of which acts individually and in combination with other factors to deleteriously affect the health of incarcerated populations.

**Table 1:** **The individual, familial and community impacts of incarceration**

	Mechanism	Individual, Family and/or Community Health Impacts
**Prison Environment**	Deprivation	• Disempowerment
		• Negative and violent confrontations during and after release
	Prison Code	• Inability to sustain case management
	Coercion	• Severance of social support
	Prison Conditions	• Poor ventilation
		• Overcrowding
		• Isolation
	Provision of Correctional Healthcare	• Stabilizing, but can:
		○ Lack quality
		○ Lack access
	Lack of Comprehensive Incarceration Programs and Discharge Planning	• Existing substance abuse or mental health issues can be exacerbated
		• Lack of support during post-release transition
**Post-Release**	Continued Loss of Social Support	• Severance of social relationships
	Enduring Stigma	• Disenfranchisement
		• Barrier to help-seeking behavior
		• Obstacle to linkage to medical and social services
		• Negative psychological adjustment
		• Disempowerment
**Macro-Policy Level**	Several Large Scale Policies that Restrict the Rights of Formerly Incarcerated Populations	• Financial insecurity
		• Inability to obtain food stamps and other health benefits
		• Unstable housing
		• Disenfranchisement
		• Disempowerment
		• Cyclical Poverty
		• Re-incarceration

### Incarceration environment

In the correctional setting, individuals are faced with a number of circumstances, each of which affects health. These include experiencing various forms of deprivation, exposure to the “prison code”, existing in a coercive and controlling environment, poor prison conditions, and the mandatory provision of healthcare.

#### Deprivation

Deprivation refers to being divested of individual rights and possessions that are afforded to otherwise “free” individuals (Sykes 1958). These deprivations might include liberty, goods and services, heterosexual relationships, security and autonomy (de Viggiani 2007). The first conceptualization of deprivation is found in Sykes’s (1958) work, in which he posited that an individual’s own sense of self-worth is negatively affected by the incarceration experience. Subsequently, many scholars have studied the concept of deprivation in prison and jails, and have also found that the deprivation of rights and freedoms adversely affects the health status of incarcerated individuals (de Viggiani 2007).

In addition, research has also demonstrated that deprivation in the prison environment leads to physical, mental, and social harm that can disempower and affect incarcerated populations to an extreme extent (de Viggiani 2007; Rhodes 2005; Shalev 2009). For instance, deprivation via isolation has been shown to have a detrimental effect on mental health (Rhodes 2005). Kurki and Morris (2001) reported that inmates often described feeling rage, anxiety, dissociation and psychosis accompanied by feelings of hopelessness while incarcerated.

However, prisons and jails have differing levels of deprivation. Research suggests that certain types of facilities such as maximum-security prisons, where deprivations are the most extreme, have greater negative health effects (Daniel and Fleming 2006; Huey and McNulty 2005; Way et al. 2005). Way et al. (2005) reported that 83% of all suicides between 1993 and 2001 in the New York Department of Corrections took place in maximum-security prisons. Scholars theorize that this link between increased deprivation and suicide is due to psychological harm and stress incurred due to segregation and isolation (Johnson 2005; King 2005; King 2006; Shalev 2009).

Finally, deprivation of social support often results due to the geographic location of many prisons. Correctional facilities are frequently located in rural areas that have little or no access to public transit and there is often no active attempt to keep a prisoner close to their home community during incarceration (La Vigne et al. 2008). Additionally, there are no financial or transportation incentives available to help keep families intact and maintain routine contact, and keeping in contact via telephone often includes exorbitant fees. In the past, for long-distance telephone calls were as high as $0.89 and usually included, on average, a $3.95 connection fee. This meant that a total of one hour of phone calls per week can lead to nearly $300 worth of phone charges each month, which creates an undue burden on families and a resulting dearth of communication (Media Justice Fund of the Funding Exchange 2009). In August 2013, the Federal Communications Commission determined that the maximum rate for a collect call made by a prisoner cannot exceed $.25 per minute. While this rate is lower than previous charges, it is still much higher than collect call fees incurred by those outside of prison. Subsequently, inmates often report a decrease in a sense of social support, which may have a deleterious impact on his or her overall well being.

#### Prison code

In addition to deprivation, inmates are often exposed to the prison code while incarcerated. The prison code rewards hyper-masculine behavior that is often the cause of negative health outcomes or re-arrest after release (Petersilia 2008). Research has explicated the specific tenets of the prison code and found the following to operate as normative behaviors in the prison environment: a tough persona, suppression and denial of fear, weakness or suffering, an aversion to collaboration with prison guards and staff, refusal to report delinquent behavior of a fellow inmate, the inclination to hide affinities toward femininity, jostling for recognition, the willingness to fight to defend one’s honor, and struggles for dominance (Trammel 2012). Furthermore, one’s ability to follow the code can also elevate or diminish status while incarcerated (de Viggiani 2007). Therefore, those who exhibit the most violent behavior occupy the uppermost social status in prison while a majority of offenders inhabit the middle stratum; Ostracized groups, such as sex offenders, occupy the lowest class (Marshall et al. 2000; Miller 2000; Archer 2002). This social hierarchy can lead to conditioned behaviors, which may result in negative and violent confrontations after release, furthering the likelihood of re-arrest (Petersilia 2008).

Additionally, as noted by Rotter et al. (2005), the behaviors rewarded by the prison code that are exhibited post–release can foster a hostile environment that interferes with community adjustment and personal recovery. These manifestations also may negatively affect the relationship between the released inmate and social service and medical providers, hindering a previously incarcerated individual’s ability to access much needed medical care. Providers may also misinterpret the signs of a challenging re-adjustment as opposition to treatment, lack of motivation to change or reintegrate, proof of individual pathology, or indications of a serious mental illness (Rotter et al. 2005). Due to potential exposure to multiple stressors during this transition period, there is increased need for the intentional development of opportunities to decompress and recover from having to constantly portray a tough exterior in the incarceration environment that some scholars like to post-traumatic stress disorder (Liem and Kunst 2013). Relatedly, Jewkes (2002) summarizes the tension between the contrasting behavioral expectations of the prison or jail environment and the community that confront individuals upon release, saying that:

[T]he tensions associated with sustaining the particular bodily, gestural and verbal codes demanded….are particularly marked, and the necessity for a deep backstage area where one can “be oneself”, “let off steam” and restore one’s ontological reserves is therefore arguably even greater than in other settings (p. 211).

Therefore, the prison environment, and by extension abiding by the prison code, may not only have deleterious effects on the mental health of incarcerated individuals, but can also have sustained effects after release via the possible inability to retain relationships with post-incarceration case managers and social support networks (e.g. family members and friends).

#### Coercion

In addition to the prison environment’s ability to reinforce a violent and counterproductive code of conduct, it can act as a source of disempowerment via coercion. Sykes (1958) posited that inmates exhibited a self-interested mode of behavior that was a result of being mandated to observe roles that were required by the prison regime and ensured their survival in prison society. Recent research has reinforced Sykes’s results and has also found that that those who are incarcerated develop disciplined and habitual behaviors and subscribe to an inflexible structure of standards that are mandated by an unbending environment (de Viggiani 2007). Relatedly, inmates may experience an alternative, incarceration-related version of what Ross (2011) refers to as “neighborhood disorder”, wherein a dangerous environment in which an individual has little control over their circumstances induces feelings of powerlessness, stress, anxiety, anger and depression.

#### Prison conditions

A related circumstance that may lead to negative health outcomes for inmates is prison conditions. As Drucker (2011) notes, due to the increasing number of incarcerated individuals, prisons are experiencing unprecedented and unanticipated problems, such as overcrowding that is associated with prison mortality (Rabe 2012). These negative prison conditions have direct effects on prison populations and their health. For instance, Drucker (2011) posits that worsening prison conditions and the increase of violent encounters lead to threats to inmates’ health and safety. Additionally, some research attributes worsened health to the built environment and design of the prison. Awofeso (2011) makes the claim that the architectural design of incarceration facilities can exacerbate conditions such as tuberculosis due to poor ventilation and crowded and shared prison cells and common spaces. Further complicating the impact of the built environment of prisons and jails, contemporaneous policies such as solitary confinement have been linked to depressive and suicidal tendencies among prisoners (Haney 2003).

Additionally, in 2011, the Supreme Court ruled in *Brown v. Plata* that the state of California was to release 46,000 prisoners because overcrowding violated the 8^th^ Amendment. The extreme overcrowding in California’s state prisons was deemed cruel and unusual punishment because prisoners were not receiving proper healthcare while incarcerated (Applebaum 2011; Newman and Scott 2013). This decision has important implications for the incarceration environment by potentially limiting the overcrowding that can occur in prisons and jails. Additionally, the importance placed on correctional healthcare provision makes clear the right prisoners have to routinized health services.

#### Correctional healthcare provision

Research has demonstrated that healthcare provided in incarceration facilities may be the only healthcare incarcerated individuals can access, and has, therefore, been deemed as better than receiving no care at all. After all, incarcerated populations constitute the only group who has the constitutional right to healthcare, and as demonstrated by the Supreme Court’s decision in *Brown vs. Plata*, not receiving proper healthcare while incarcerated is legally considered cruel and unusual. Wacquant (2002) states that healthcare in prison or jail facilities cannot be described as “distortive and wholly negative” because it may act as a “stabilizing and restorative force” (p. 388), especially for those with many barriers to accessing healthcare in the community. Indeed, most correctional facilities do provide screening for infectious diseases such as HIV. However, a fair amount of research has highlighted the need for better access to and quality of care within incarceration facilities due to the increased likelihood of pre-existing chronic and infectious diseases within prison and jail populations (Magee et al. 2005; Massoglia 2008). Receiving poor care within a prison or jail can still negatively affect individuals’ health and may lead to worsened health outcomes (Hatton et al. 2007). For instance, Brinkley-Rubinstein and Turner (2013) found that HIV positive inmates often experienced a delay in medical treatment and low quality of care while incarcerated. And, even though access to care is guaranteed in correctional institutions, Mallik-Kane and Visher (2008) found, in a nationally representative study of prisoners that many with pre-existing medical conditions did not receive treatment while incarcerated. Relatedly, even though research shows that a large number of those who are incarcerated either have mental health and/or substance abuse issues, only one-fourth of incarcerated populations received treatment for these conditions while incarcerated (Petersilia 2008). Whereas recent research has highlighted some specifics about healthcare provision in correctional facilities, much is still not known about access and quality of healthcare in jails and prisons. Research of this variety is increasingly relevant, in part because recent healthcare reform provides an opportunity to offer more consistent and expanded health coverage to individuals who are at most risk of experiencing incarceration.

#### Lack of educational and discharge programming

Finally, in addition to the impacts of the incarceration environment and healthcare provision there is a lack of educational and discharge planning programs in correctional settings. While prison education programs once were widely available, the elimination of prisoner eligibility for Federal Pell education grants in 1994 caused participation in postsecondary correctional education programs to decrease 44% (Crayton and Neusteter 2008). Most prisons still have correctional education programs, but only one-third of prisoners who are released will have participated in some type of work training or educational programming while incarcerated (Crayton and Neusteter 2008; Petersilia 2008). Additionally, the rate of participation in education programs offered in correctional facilities has not grown proportionate in comparison to the prison population as a whole (Western et al. 2003). Rather, the percentage of participation in educational programs has gradually decreased over the years (U.S. Department of Justice, Bureau of Justice Statistics 2007; Crayton and Neusteter 2008; Harlow 2003). This decline is relevant in that a large body of research demonstrates the efficacy of educational programming in significantly decreasing the likelihood of recidivism (Chappell 2004; Flinchum et al. 2006). Additionally, incarcerated individuals are much more likely to have lower levels of education than the general population, but incarcerated populations tend to have higher literacy scores than their counterparts in the general population. This points to the fact that for systematically disenfranchised populations, prisons may be the primary route to obtaining educational opportunities. Upon release, such educational experiences are important predictors of well being, as socioeconomic status is one of the strongest social determinants of health. However, even though over 93% of correctional leaders support the offering of educational and vocational opportunities in prisons, the increasingly punitive carceral environment has led to the deterioration of educational opportunities for correctional populations (Tyler et al. 2006).

Relatedly, there is a dearth of discharge planning services, including a lack of aid in linkage to medical services, and assistance finding employment and stable housing for individuals nearing release (Petersilia 2008). Such assistance is extremely important to successful reintegration as the first six months after release is when individuals are at the most risk of re-incarceration (Petersilia 2008). Moreover, transition support has been positively associated with increased healthcare access after release from prison or jail (Wenzlow et al. 2011).

### Persistent post-release mechanisms

The negative health effects of incarceration do not end after release. In fact, they are only compounded and made worse by the post–release experience, which often includes the continued effects of the involuntary loss of social support, and the enduring stigma attached to having a criminal record.

#### Continued loss of social support

A large amount of research has investigated the importance of social support and ties, especially within vulnerable populations (Karb et al. 2013; Knowlton 2003). Social support has been shown to mediate engagement in risky behavior and serve as a facilitator of individual and collective empowerment (Gabriel 2007; Lauby et al. 2012). Additionally, research has demonstrated the link between social support and health, indicating that higher levels of social support lead to more positive health outcomes (Sarason et al. 2010). However, in contrast, several policies regarding prisons actively sever social relationships. This loss of social support during incarceration can extend to the post-release period and can negatively affect health. For instance, Khan et al. (2011) found that engagement in primary partnerships might decrease sexual risk-taking among men involved in the criminal justice system but that 55% reported that their relationships ended during incarceration. A lack of social support can also negatively influence reintegration after release. Binswanger et al. (2012) found that lack of social support resulting in feelings of isolation often led to an increased likelihood of a reluctant return to alcohol and drug use.

#### Enduring stigma

Relatedly, individuals who have been incarcerated are also likely experience stigma or discrimination. Stigma refers to unfavorable approaches, views, and, at the macro-level, policies that are directed toward people who belong to a shunned or socially marginalized group (van Olphen et al. 2009). Goffman (1963), characterized stigma as an attribute that makes a person undesirable within specific social spheres. Formerly incarcerated individuals are deeply stigmatized due to the negative association with having been imprisoned and, as a result, are marginalized and excluded from myriad federal assistance programs and access to many types of employment (Petersilia 2008). However, stigmatization not only leads to marginalization through various policies, but also has the potential to weaken ties and social support received from law-abiding citizens (Petersilia 2008). Thus, the labeling of an individual as “delinquent” results in further disenfranchisement and propensity to engage in criminal activity (Johnson et al. 2004).

Stigma can also have a major effect on health (Hatzenbuehler et al. 2013). For instance, Earnshaw et al. (2013) found that stigma was a significant indicator of physical health for HIV positive individuals. Additionally, stigma can act as a stressor that may be associated with negative psychological adjustment, help seeking behaviors, and access to medical and social services (Brinkley-Rubinstein and Turner 2013; Masuda et al. 2012; Vanable et al. 2006). This is particularly salient for incarcerated populations who face continual, multi-level stigma long after they are released, which affects their ability to reintegrate into the community and access multiple systems of support. As a result, the process of disenfranchisement incurred via multiple forms of stigma has the ability to worsen individuals’ health or exacerbate existing health concerns.

### The impact of policy on social environment after release

In addition to the health impact of the correctional environment and enduring post-release mechanisms, incarceration-related policy can also have a significant impact on well being. In the past decade, rehabilitation services and policies to help inmates reintegrate into the community have disappeared, whereas the legal and practical barriers to reintegration have increased (Petersilia 2008). Research has shown that, as a result of the tougher political stance towards crime, a restriction of the rights of ex-prisoners has proliferated (Cnaan et al. 2008; Clear 2007; Wacqant 2010).

Travis (2002) referred to these restrictions as “invisible punishments,” as they are indirectly and continually punitive toward ex-inmates far after their initial release from incarceration. In regards to the policies, he commented:

Over the same period of time that prisons and criminal justice supervision have increased significantly, the laws and regulations that serve to diminish the rights and privileges of those convicted of crimes have also expanded. Yet we cannot adequately measure the reach of these expressions of the social inclination to punish (p. 16).

These “invisible punishments” inhibit successful transition, affect wellbeing and macro-level policies mostly aimed at those convicted of a felony offense.

#### Lack of access to jobs

Those who are convicted of a felony are restricted from serving in the military, having a government position, or obtaining a number of permits and licenses (Iguchi et al. 2005). In addition, employers are increasingly resistant to hiring ex-inmates and are more often requiring background checks during the hiring process (Petersilia 2008). This is important because the ability to find employment post-release is essential to successful reintegration. Geller et al. (2006) found that formerly incarcerated men experience a 14 to 26% decline in hourly wages compared to their earnings prior to incarceration. Relatedly, Pager (2007) found that those who had a criminal record were less likely to obtain an interview after disclosing their criminal history. Lack of formal employment opportunities often pushes individuals into participation in the informal economy. Brinkley-Rubinstein and Turner (2013) found that informal jobs such as cleaning a neighbor’s home, washing cars, or even sometimes dealing drugs were the only types of employment available to formerly incarcerated individuals. These types of employment opportunities may negatively affect health because they do not offer health insurance or other health promoting benefits and they may be dangerous (e.g. dealing drugs) increasing the likelihood of harm while performing their associated tasks.

#### Decreased availability of health benefits

Formerly incarcerated individuals may also lose access to various federal benefits. When convicted of a felony, individuals are often unable to collect food stamps or social security insurance either temporarily or permanently, dependent on the state in which they reside (Iguchi et al. 2005; Raphael and Stoll 2009). Additionally, while low-level offenders may not lose eligibility for benefits, those incarcerated for more than one month may experience a termination or suspension of benefits while they are incarcerated and the reenrollment process can be cumbersome. This lack of access to benefits that promote health may have a detrimental effect on an incarcerated individual’s ability to reintegrate into their community after release and may delay individuals with serious illness from seeking medical care.

#### Lack of access to public housing

Obtaining stable housing after release is also often difficult. In fact, parole officers have indicated that finding housing for formerly incarcerated individuals is one of the largest challenges to successful transition (Petersilia 2008). Procuring housing is made more difficult by the “One Strike and You’re Out” legislation passed by Congress in 1996, which gives federal housing authorities the discretion to decide whether to allow those with a drug or alcohol related offense and their families to access federally subsidized housing (Iguchi et al. 2005). Also contributing to untenable living conditions following release is that parole conditions often prohibit ex-inmates from living with other individuals who have been involved with the criminal justice system (Petersilia 2008). This may have the potential to impact family structure in that an incarcerated parent or partner may be unable to obtain housing in which an entire family may reside. A newly released incarcerated family member may be restricted from living with loved ones due to other family member’s prior involvement with the criminal justice system.

Despite the importance of stable housing as a factor for successful community re-integration, there is very little scholarship that examines the experiences of homelessness after release. However, Metraux and Culhane (2004) indicate that 11.4% of formerly incarcerated individuals in New York entered a shelter within two years of release. Additionally, 62% of formerly incarcerated individuals in New York City spent their first night post-release with relatives, and a year after initial release only 10% were paying rent on their own home or apartment (La Vigne et al. 2004; Visher and Courtney 2007). This is important to prisoners’ health because lack of stable housing and homelessness has been associated with poor health outcomes and complicates the delivery of adequate healthcare (Wright 2010).

#### Lack of financial support for higher education

Barriers are also in place that prohibit access to federal aid for higher education. The Higher Education Act (1998) states that those with drug possession convictions are no longer eligible for federally supplemented aid for one year after a first conviction, two years after their second conviction, and indefinitely after their third. This penalty is even harsher for an individual who is convicted of selling drugs. If convicted for selling drugs, the offender is ineligible for education assistance for two years after their first offense and completely ineligible after any subsequent arrest. However, it must be noted that there are provisions in place that allow reinstatement of education benefits after evidence of drug rehabilitation and a certain number of clean drug tests. Nonetheless, restrictions regarding the ability to finance higher education act as a major hindrance to upward social mobility for this particularly vulnerable population. These barriers are even more important in light of research demonstrating the strong relationship between health and education (Miech et al. 2011).

#### Lack of right to vote

Additionally, despite some reforms in the last 15 years to restore the right to vote, in 2008, nearly five million individuals were unable to vote in the presidential election (King 2008). Those who are ineligible to vote live in 35 states wherein individuals on parole, probation or who have served their sentence in its entirety are unable to exercise their right to vote. Due to the disparate rate of incarceration of minority populations, Bowers and Preuhs (2009) estimate that over 10% of all African Americans and 1 in 8 of all African American men are ineligible to vote. This lack of voter eligibility diminishes the political power of particularly vulnerable, minority and at-risk communities, and stems their ability to organize around important community and societal health-related issues, such as HIV/AIDS, access to care, increased health insurance availability and coverage and other related public health issues. Relatedly, research has shown that civic engagement can have a direct and positive effect on health outcomes (Murayama et al. 2012).

#### Compounding impact of cyclical poverty

All of these incarceration related mechanisms in combination perpetuate the cyclical feedback loop of poverty. In 2009, there were over 43 million individuals living in poverty in the U.S. This represents a proportional increase of the total population: from 13.2% in 2008 to 14.3% in 2009 (United States Census Bureau 2010). This percentage becomes even more alarming when the percent of poverty is stratified by race. In 2009, nearly 26% of African Americans fell under the poverty line, an increase from 24.7% in 2008 (United States Census Bureau 2010). While poverty does not create crime, those with the least amount of economic resources are the most likely to end up in prison and jail (Lyons and Walsh 2010). As Kurgan (2013) notes the communities with the highest rate of incarceration almost perfectly overlaps with the most impoverished neighborhoods in most major metropolitan cities. Additionally, while those who are most likely to be incarcerated are at increased risk of living in poverty, incarceration itself is a risk factor for impoverishment. As previously expounded upon, formerly incarcerated individuals often cannot obtain employment after release and may find it hard to access jobs that offer training and pay schedules that have predictable pay increases. This is important because those who are most economically deprived are also the most likely to be unhealthy. Those who are impoverished are more likely to have more prolonged illnesses and more recurrent and severe disease complications, thereby making greater demands on the healthcare system (Woolf et al.^2006^). This often inescapable feedback loop of incarceration and poverty not only diminishes health but also leads to less successful rates of reintegration after release.

#### Re-incarceration

Research that evaluates the frequency of recidivism among released inmates has found that between 43-45% return to jail or prison within three years of their initial release (Pew Center on the States 2011). Additionally, the re-arrest rate is growing, and is 5% higher than it was in 1983 (Petersilia 2008). There is no prevailing evidence that incarceration and subsequent re-incarceration reduce crime. In fact, there remains no correlation between crime rates and incarceration rates (Alexander 2010). However, repeated imprisonment can negatively affect one’s health status in that they are continuously exposed to the multiple stress-inducing mechanisms of incarceration.

### Familial and community impacts

While all of the mechanisms of incarceration affect the individual who is currently or formerly incarcerated, many also cascade, affecting families and communities. For instance, as stated previously, incarceration facilities are often in very remote places that can make it difficult to sustain familial relationships because of lack of availability of public transport (Comfort 2008). Specifically, Naser and Visher (2006), when interviewing family members of incarcerated individuals, found that the number one cited barrier to visiting was the distance to the correctional facility. Additionally, 80% of those in state prisons for non-violent offenses report having at least one prior conviction, indicating continued disruption of relationships between family members because the incarcerated individual is cycling in and out of prison or jail (Durose and Mumola 2004). Research has also shown that incarceration can have multiple effects on family members. The immediate effects of the loss of a partner or parent to incarceration can include feelings of shame or stigma, loss of financial support, weakened ties to the incarcerated individual, poor school performance, increased likelihood of delinquency, and increased risk of abuse or neglect (Geller et al. 2009; Geller et al. 2011; Phillips et al. 2006; Schwartz-Soicher et al. 2011; Wildeman and Western 2010;). Additionally, the long term effects of incarceration include parental-child conflict, negative perceptions of police or other representatives of the criminal justice system, delays in child development, increased levels of aggression in children and increased dependency or inability to cope with various stressors and trauma (Geller et al. 2012; Wakefield and Wildeman 2011; Wildeman and Western 2010).

Many of these short- and long-term consequences are also risk factors for incarceration. As such, children who have an incarcerated parent are more than six times as likely than other children to experience incarceration at some point in their lifetime (Hairston 2007). Therefore, Wildeman and Western (2010), posit that for certain subsections of the population being incarcerated happens so frequently and has such a deleterious effect on children and families that incarceration singlehandedly creates more inequality and more future likelihood of crime. Thus, children whose parents have experienced incarceration have an extremely increased likelihood of becoming incarcerated themselves and being exposed to the multiple mechanisms of incarceration that act as a catalyst to worsen health.

Finally, it is important to consider the community level impacts of incarceration. The theory of coercive mobility posits that incarceration, in aggregate, can have inadvertent consequences on the community level (Rose et al. 2003). Building on this theory, Rose et al. (2003) found that mass incarceration in specific communities impaired the human capital of non-incarcerated community members. For example, parents were often more stressed and children were sometimes hungry, had truancy problems, were disciplined less by caregivers and, sometimes, participated in criminal activities. Additionally, residents of communities with high levels of incarceration reported feeling oppressed and abandoned by the government (e.g. lack of attention related to addressing community problems) (Rose et al. 2003). This is corroborated by Wacqant (2010) who elucidates that the workfare revolution resulted in the elimination of a majority of services that were previously provided in low-income neighborhoods.

However, the cumulative impact of incarceration is hidden to a majority of Americans who do not reside in communities with concentrated populations of incarcerated individuals. Low income, African Americans are disproportionately incarcerated and, as such, the collateral consequences of incarceration impact these communities more than others (Clear 2007; Dumont et al. 2013; Wacqant 2010; Mauer and Chesney 2002). Research shows that incarceration may lead to higher crime rates rather than act as a deterrent and, as such, make communities more vulnerable and further socially disintegrated (Sampson and Loeffler 2010). Kurgan (2013) highlights the work of the Justice Mapping Center (2007) that demonstrated that in New York 75% of the entire prison population came from only seven neighborhoods in New York City. These seven neighborhoods are comprised of mostly impoverished, minority populations and due to disinvestment of other civic infrastructure the criminal justice system has become the only governmental representative. Thus, there are extreme opportunity costs to education, housing, and health. In order to stem intergenerational incarceration and buffer the familial and community level effects of incarceration, future research must examine these higher order outcomes, focusing on how particular intervention and prevention efforts may address incarceration’s harmful effects.

## Implications

As the rate of incarceration remains at historically high rates, the social response has worked to further disenfranchise already vulnerable populations. Additionally, a majority of interventions and programs that are targeted at incarcerated populations are implemented solely at the individual level. For instance, routine and often implemented programs include linkage to medical care services or individually focused case management (Draine et al. 2011; Guydish et al. 2011; Goldstein et al. 2009). However, due to the multi-level impact of incarceration, the focus of interventions and programs must shift to foster an approach to reintegration that successfully improves the conditions of the offender, both during incarceration and following release.

## The need for multi-level model interventions to mediate the effect of incarceration on health

While more interventions and programs that address micro *and* community-level issues relevant to incarceration are needed, there are examples of successful individual-level programs that aim to provide solutions to various problems caused by incarceration. These interventions occur both inside and outside the prison or jail and seek to improve outcomes affected by both incarceration environments and the transition back into the community after release. For instance, many successful interventions have utilized motivational interviewing, peer driven case management and other behavioral change strategies (Goldstein et al. 2009; Farbring and Johnson 2008). However, it should be noted that this type of intervention explicitly works only on the individual level and ignores the structural and institutional role in incarceration. Therefore, individual-level interventions such as motivational interviewing should be paired with a community level or policy level intervention to be most effective.

Holistic approaches that address multiple levels (e.g. individual, family and community) and that are focused specifically on the ways in which incarceration affects health are needed. These interventions should focus on the structural determinants that affect individuals on each socio-ecological level and, thus, ideally must also be multidisciplinary and include partnerships across sectors and disciplines. There must be a focus on the ways in which macro-level policies manifest in individual behaviors and do not be have to be primarily intended to directly affect health. Instead, interventions should also focus on the social conditions that have been demonstrated to indirectly affect the health of incarcerated individuals such as stigma or loss of social support.

Subsequently, interventions targeting incarcerated individuals must also acknowledge the intersectional nature of the inequality that is present within this population. Interventions that solely address substance abuse, mental health issues, housing issues or any of the other barriers to successful reintegration cannot effectively ensure success upon release or improved health outcomes. The additive nature of these barriers requires cross-governmental and organizational collaboration. For instance, for multi-level, holistic interventions to work, involvement of both public health entities and criminal justice agencies is required. Additionally, discharge planning should begin well in advance of an individual’s eventual release and should be comprehensively provided for at least six months since this time period is when formerly incarcerated individuals are the most likely to be re-incarcerated (Petersilia 2008).

Action-oriented and community-based participatory interventions and approaches may be effective avenues for the application of intersectional and socio-ecologically-influenced strategies aimed at mediating the effect of incarceration on health. The inclusion of the community during the conception, design, and administration of intervention efforts lends voice to community members to determine which issues they think are the most important to address, and empowers individuals to create answers to their own concerns (Cornwall and Jewkes 2010). Engaging the community via participatory approaches can lead to more effective program implementation and design as well as inform relevant policy decisions that may minimize the impact of incarceration on health (Choudhry et al. 2002; Ganann 2013).

## Suggestions for policy change

The drastic increase in the number of incarcerated individuals can be explained in large part due to various policies requiring harsher sentencing of drug-related offenses. Additionally, federal restrictions resulting imposed on the procurement of federal aid by a drug offender has made post-release reintegration increasingly difficult. The loosening of these policies is required if we, as a society, are to end the era of astoundingly high rates of imprisonment and move to a more effective model of rehabilitation.

Over the last three years there has been a gradual, slight decline in the number of inmates in state and federal prisons (Golinelli and Carson 2013). Recent policy shifts may, currently or in the future, aid in shifts in the carceral landscape. The Patient Protection and Affordable Care Act (PPACA) will soon ensure health insurance coverage for all individuals at or below 138% of the federal poverty line. Approximately 10 million individuals cycle out of the criminal justice system each year and make up a substantial proportion of the 16 million individuals who will be eligible for Medicaid coverage via the PPACA beginning in January 2014 (Santoro 2013). Additionally, the PPACA mandates coverage of behavioral healthcare and substance abuse services. This has the potential to substantially impact individuals most at risk of incarceration in that a large number of all inmates report having mental health issues or substance dependency (Petersilia 2008). Finally, the PPACA is an important link to extend the stabilization that correctional healthcare may provide and maximize the investment that local and state governments make in correctional healthcare provision. However, as Phillips (2012) notes, the impact of the PPACA is highly reliant on whether states decide to expand beyond the federally mandated minimum requirements, the level of engagement in outreach efforts to make individuals and organizations aware of the benefits of the PPACA, state level coordination efforts between criminal justice and other agencies and whether states capitalize on expanded coverage for mental health and substance abuse treatment. Thus, while the full impact of the PPACA is yet to be determined, its potential for improving health is extraordinary. Additionally, some have posited that due to the increased coverage for behavioral health and substance abuse treatment that incarceration and re-incarceration rates will possibly decline (Phillips 2012).

Relatedly, the Obama administration recently released the 2013 Blueprint for Drug Policy that places greater importance on incarceration alternatives such as drug courts and probation programs aimed to reduce incarceration rates. The Blueprint also encourages the use of community-based programs designed to address substance use, crime and incarceration by re-directing law enforcement attention to more serious offenses. This policy shift has the potential to divert over 100,000 would be prisoners away from incarceration. The efficacy of alternative to incarceration programs such as drug and mental health courts is demonstrated in the literature. For instance, Mitton et al. (2007) found that a community-based alternative to incarceration for mentally ill offenders reduced justice system complaints, charges and court appearances between 84% and 91%. Additionally, a meta-analysis of analyzing 92 evaluations of drug court programs found that the average drop in recidivism was from 50% to 38% for participants (Mitchell et al. 2012).

Finally, the PPACA will also provide the opportunity for states to find considerable savings in their correctional budgets as many more individuals who are most likely to become incarcerated will have access to insurance and, subsequently, increased access to federally subsidized care. Therefore, justice reinvestment programs, aimed at crime reduction and community re-investment, may be a strategy worth considering. Justice reinvestment is an approach to decrease incarceration rates and related criminal justice spending, and reinvest funds in tactics that can decrease crime and strengthen communities. States and local entities engaging in justice reinvestment collect and analyze data on what motivates crime, pinpoint and execute new programs aimed at community change and measure the efficacy of any new justice reinvestment oriented intervention (Lachman and Neusteter 2012). Currently, there are ongoing justice reinvestment projects in Texas, Minnesota and North Carolina. However, as Clear (2011) points out justice reinvestment should not only focus on spending reduction and instead be concerned primarily with justice. As such, justice reinvestment efforts should be guided by a restorative justice theoretical framework (Clear 2011; Maruna 2011). Bazemore and Maruna (2009) define restorative justice as “'doing justice’ by repairing the harm caused by crime in a non-adversarial process that invites offenders to 'take responsibility’ rather than simply take their punishment” (p. 376). While some of the justice reinvestment scholarship is situated within larger restorative concepts, a general recognition of the need for justice, and thus holistic and long-term reinvestment, in historically disadvantaged communities is missing. (i.e. providing access to community based programs that address various social determinants of health). State savings derived from federally supplemented healthcare for incarcerated populations present an opportunity to implement restorative guided justice reinvestment programs for communities most affected by incarceration.

## Suggestions for future research

In the future, continued research is needed to evaluate the impact of the various mechanisms of incarceration on health. Furthermore, there is an exceptional void in the amount of longitudinal research that examines the relationship between health and incarceration. Longitudinal and in-depth research can elucidate the ways in which incarceration affects individual, familial and community health in the long-term, leading to a better understanding of the interventions that are most needed. Additionally, research is needed that tests the hypothetical heuristic path model put forth in the present paper to better understand if and exactly how the mechanisms presented combine to negatively impact the health of individuals, families and communities. Further investigation is also needed to determine if the type of correctional facility or if any of the mechanisms of incarceration affect health more or less than others. Finally, an added contribution to the health and incarceration literature would be research at the macro-level that compares more liberal incarceration policies (in states such as in Vermont and Maine where voting rights are never restricted) and more limiting policies in order to evaluate the differences in the general health of prison populations.

## Conclusion

While rates of incarceration continue to be near historic highs and the literature exploring the relationship between health and incarceration proliferates, it is important to understand the specific mechanisms through which incarceration impacts health status. This paper has attempted to delineate more clearly these mechanisms and their impacts on those most likely to be incarcerated, their families and communities. Using a model undergirded by theoretical frameworks that emphasize the cumulative effect of disparity and focus on structural approaches to change, it is suggested that multi-level approaches to transformation are undertaken. Recent Supreme Court decisions (*Plata v. Brown*) and advances via healthcare reform have the potential to positively impact healthcare access of both incarcerated and formerly incarcerated individuals. However, a more drastic policy paradigm shift is needed to address the collateral consequences of incarceration on virtually all areas of incarcerated individuals’ lives that cascades to impact the communities in which they come from and, eventually, return.

## Authors’ original submitted files for images

Below are the links to the authors’ original submitted files for images. Authors’ original file for figure 1 Authors’ original file for figure 2
