# Supplementary figures and images for: Incarceration as a catalyst for worsening health
Source: Health Justice. 2013 Oct 24;1:3. doi: 10.1186/2194-7899-1-3 (PMC5151791; doi:10.1186/2194-7899-1-3)

**Figure 1: Conceptual Model of Incarceration’s Multi-level Impact**


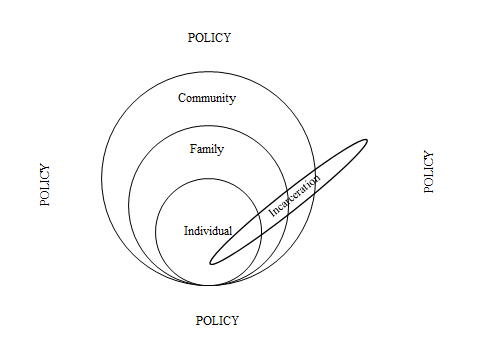

Supplement: Supplementary file 1 — Authors’ original file for figure 1 [file 40352_2013_17_MOESM1_ESM.docx]

**Figure 2: Incarceration as a Catalyst for Worsening Health**


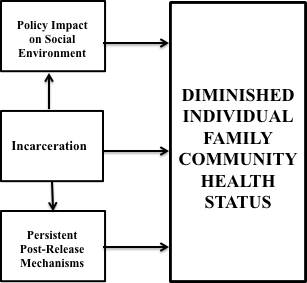

Supplement: Supplementary file 2 — Authors’ original file for figure 2 [file 40352_2013_17_MOESM2_ESM.docx]
